# Electro Galvanism

**Published:** 1850-11

**Authors:** Thos. D. Washburn

**Affiliations:** Grayville, Ill.


ARTICLE I.

*Electro Galvanism.*—A paper read before the Æsculapean Society, by THOS. D. WASHBURN, M.D., of Grayville, Ill., ordered to be published by the Society.

Among the many innovations which have quietly forced themselves into the confidence of the profession, I know of no one presenting stronger claims to our consideration than that of Galvanism, or Electro-Magnetism.

1st, From its *efficacy* as a medical agent.

2d, Because it reaches a variety of cases hitherto almost irremediable, and many of the most painful nature to the patients, and harrassing to the medical attendant.

3d, Because of its eligibility of application.

It is some half century since Galvanism was first discovered; its application to medicine is of much more recent date. About twenty-five years since, it began to attract general attention, and numerous experiments began to be instituted and new features, laws, and phenomena, have continued to be developed up to the present time.

It will hardly be worth while now to trace the many and various experiments by which it has arisen from a strange mystery to a beautiful science; our object is to show its influence on the vital system, and its value as a therapeutic agent.

"The galvanic current when brought to act upon the living body is capable of producing three orders of effects.

"1st, It produces peculiar sensations.

"2d, It determines muscular contractions.

"3d, It is supposed to influence the organs of secretion."

It matters not whether the galvanic current is applied directly to the muscle or part through which you wish to transmit it, or only to the *nerve*, which supplies the part, the effect is about the same: but it should be borne in mind, in reference to the influence of galvanism on the muscular system, that a shock is felt and convulsions produced, not only when the circuit is completed, but also at the moment when it is broken, and during the *maintenance* of the circuit, these effects are *not* perceived.

Here it may be well to mention the comparative value of the two agents, Electricity and Galvanism. "The discharge of a Leyden jar and a Galvanic battery produce sensations so similar as to render it probable that they affect the living body in the same way." But Dr. Bardsley and Mr. Donovan, of Dublin, contend that Galvanism is much the most efficient agent, and conceive it acts more deeply within the organs, while Electricity acts more superficially; and at the school of medicine, in Paris, the commission which reported on the subject, found reason to conclude that the "effects of the battery penetrate and effect the nervous and muscular structures more deeply than ordinary electric machines;" at any rate the Galvanic battery possesses advantages which will always give it preference.

1st, The quantity of Electricity it sets in motion is vastly greater, and yet, at the same time can be graduated to a minimum dose with perfect ease.

2d, You can apply it with facility to any part or organ, without affecting the whole body, as for instance in partial paralysis, aphonia, amaurosis, and deafness.

3d, You can bring it into action in all weathers without diminishing its power.

In regard to the effect of Galvanism on the secreting organs, some interesting experiments were made by Dr. Wilson Phillip, which I will mention. "The par vagum, or eighth pair of nerves, which are chiefly distributed to the stomach and lungs, were divided in the necks of several rabbits shortly after having fed on parsely. Their respiration was thus rendered laborious, nausea and fruitless attempts at vomiting supervened and the animals finally died, apparently of suffocation. Upon opening their stomachs, also, the parsely was found quite unaltered, from which he concluded that the secretion of the gastric juice had been suspended.

The same experiment was then performed on other rabbits, with this difference, that galvanic currents were sent to the stomach by applying one of the poles of a small pile to a slip of tin foil wound round the lower ends of the divided nerves, and the other pole to a disc of silver laid upon the epigastrium. In all these cases dyspnoea and tendency to vomiting were wanting, and the animals being killed, after the currents had been continued for twenty-six hours, the parsley was found perfectly digested, and the stomach of each exhaled the odor peculiar to this organ during digestion." From experiments such as these, Dr. Phillip justly concludes that the secretion of the gastric juice was under the control of the nervous influence; but whether this is identical with the power generated by galvanic combination perhaps has not been sufficiently proven. Some have even gone so far as to affirm, that the nervous fluid was not only identical with galvanism, but that "animal heat was generated by currents of electricity ever circulating through the arterial branches of the sanguiferous system;" but the demonstrations of Liebig, I think, must for ever settle the question of animal heat.

Previous to the introduction of the modern Electro-Galvanic machine, Prevost and Dumas suggested the galvanic pile for for the purpose of breaking down the material which compose urinary calculi, but their experiments though partially successful were so difficult of practical application that other mechanical means have taken the place of it.

The next application was in the treatment of Asphyxia, whether proceeding from strangulation, drowning, narcotic poisons, the inhalation of noxious gases, or simple concussion of the cerebral system.

In all these cases the interrupted current is the proper one, and should be transmitted along the course of the nerves. M. Goudret was among the first to direct attention to this point by certain experiments on animals. "A Rabbit which had been to all appearance killed by a few blows on the head, was perfectly recovered by a succession of shocks continued for half an hour. Similar experiments were also performed by Mr. Apjohn. and Magendie states that himself, Pouillet and Roulin had repeatedly succeeded in recovering by means of the pile, rabbits asphyxiated by submersion in water for more than a quarter of an hour, and adds the important remark that *patience* on the part of the operator is particularly indispensable, inasmuch as in cases finally successful reanimation was often not achieved for full thirty minutes.

It is highly probable that it would be equally successful in cases of narcotic poison. But I must not forget to make a distinction pointed out by Mr. Apjohn in his able article in the Cyclopedia of Practical Medicine, on the subject, viz.: that Galvanism, flowing in a continuous current, produces a condition on the part or system, opposite to that generated by the interrupted current; the former acting as a sedative and producing relaxation; the latter as a stimulant and tonic. The former should be transmitted along the nerves in a direction *contrary* to their ramifications, the latter *with* the *course* of the nerves. Some discrimination is consequently necessary in appropriating the one or the other according to the character of the disease.

In cases of Tetanus, Epilepsy, and some forms of Neuralgia and Asthma, the continued current is the one indicated, while Asphyxia, Paralysis, Chronic Rheumatism, and other affections, the result of debility or want of power, will receive most benefit from the stimulus of broken currents.

The Electro-Galvanic or Electro-Magnetic machines, as they are termed, which have become one of the essentials to the complete outfit of a Drug establishment, are all supplied with a vibrating or revolving armature, which breaks the current and produces by its alternate action a succession of shocks; but the more common way of using the continuous current has been to place some moistened bits of sponge or linen where you wish to transmit the current, and cover one with a disc of silver, the other with copper, and unite the two points by a copper wire, or the galvanic trough may be used and connected to these points by the same discs.

In part 18th of Braithwaite, we find a communication by Surgeon Wells, who witnessed the administration of Galvanism at the Civil Hospital of Corfu. He describes the apparatus as consisting of an "oval plate of zinc from 2 to 4 inches in diameter, to the back of which a flexible wire of pure silver is soldered, and at the other extremity of this wire a plate of pure silver of the same size; the length of the wire is unimportant, varying according to the distance of the plates."

With this apparatus Ulcers, Fistulæ, fungous growths, and certain nervous diseases, were successfully treated. Some dozen rules are given for its application, for which I shall have to refer the curious to his article.

The ordinary machine of the shops, sold for medical purposes, is made up of a battery consisting of a cylinder cup of copper some 4 inches in diameter and some 5 or 6 inches in height, within which is placed a cylinder of zinc kept from contact with the copper by two small wooden ears; attached to each cylinder is a *small* metal cup, in which the connecting wires are inserted and, passing to similar cups on the base board, connect with a helix (a large coil of insulated wire) in which is placed a piston of soft iron rods, which, by moving in or out, regulates the force of the shock. The helix is also connected to an upright magnet wound with insulated wire, upon which the armature plays. A strong solution of sulphate of copper placed in the cylinders after the connexions are made with the metal cups, generally starts the machine in a few minutes, and it is ready for application. The helix is usually surmounted with a grooved piece of metal, and if one of the connecting wires is taken from the base board and passed over this, a slow, heavy shock is given.

A good Battery with the necessary apparatus can be purchased at the East for $10, giving shocks as severe as a strong sound man can withstand.

There are other more complicated machines which sell from $20 to $25.

With the action and use of these machines I have been familiar for the last six years, and have seen them applied in various cases; in some with marked benefit, in others with decided success, and in no case have I ever known it prove deleterious, though I think there are cases which might be aggravated by its application.

Of the class of diseases in which Electro-Galvanism has been found most serviceable, that of paralysis is perhaps the most prominent, and even here it must not be applied indiscriminately; in a recent case of Hemiplegia, the result of extravasation or effusion, the immediate application of this agent would be injurious, while after absorption had taken place in part or full and the nervous system remains paralyzed from disease, the stimulus by the machine would be highly useful in restoring the nervous energy and imparting tone to the affected part.

All cases of Paralysis, the result of organic disease either in the brain, spinal column, or ganglionic system, would not probably be improved by the excitement of Galvanism; but those of a purely functional kind, as general or local Paralysis, arising from exposure to cold, absorption of lead or other poisons, want of nutrition, and the sequelæ of violent and protracted disease, these and some varieties of deafness and amaurosis would be essentially benefited by the proper application of Electro-Magnetism.

Nor are these cures performed by any *magic* influence, or any *unseen* and *mysterious* agent, shut out from all approach to science and reason. The merest tyro can ascribe a satisfactory explanation of the "modus operandi." The Galvanism traveling along the nerves excites muscular contraction, this increases the circulation, determines more arterial blood and through *it* imparts warmth, health and vigor to the part.

In the 14th No. of "Braithwaite," Dr. Golding Bird reports some half dozen cases of different forms of Paralysis treated by Electro-Galvanism with remarkable success, and at the close of the article adds, "In the great majority of forms of Palsy above described, it is indubitably, in some, the actual curative agent; in all, it expedites and aids the cure; in none is it injurious."

Mr. J. C. Christophers, in the *Lancet* of '48, narrates several cases of interest, two of which I take the liberty of introducing to your notice. The first was a case "of Chronic Rheumatism of three years' duration, and of most intractable character, the patient having undergone long courses of medical treatment, besides sea-bathing and Homœopathic treatment. First, says he, I passed a current down the spine for half an hour or longer, then from the hand to the spine an hour. At the end of a few weeks she began to experience a feeling of warmth in the hands, and their strength gradually returned, so that by degrees she resumed her labors, continuing the Galvanism daily as before. Her hands have almost, if not quite, acquired their former power, (the dislocation of course remains) though she is still under treatment for the feeble condition of her ankles and knees, and is galvanized three times a week with increasing benefit to both.

"Last winter this patient was confined entirely to her bed, this winter she has not kept her bed a day; moreover regularly followed her occupation (that of Laundress) through a very severe season, and surrounded by circumstances eminently tending to produce an accession of the disease."

Case 2d, "A fine well made man, in the prime of life, was attacked some years since, with severe pain about the hip joint, extending to the knee, which was at first treated as rheumatic by drugs, counter irritants, &c., and by the introduction of needles at short intervals from the hip down to the knee.

No benefit was thence derived, and he was obliged to throw up his employment. He consulted several practitioners, without obtaining relief, and amongst others myself. He complained of great pain in the buttock and knee joint.

The affected limb was two inches shorter than the other, much less in circumference, flabby and cold; the power of motion was perfect, though sensation was evidently impaired. Here was another intractable case, in which a host of remedies had been tried without success; still there were circumstances about it that induced me to add Electro-Galvanism to the number, and with these results,—that the pain gradually decreased, and the limb regained its size, strength, firmness and temperature, and that the patient is now able to earn his living by following his occupation."

The chronic character of these cases, and their obstinate resistance to treatment, render them unusually instructive. Other cases could be mentioned; several have fallen under my own observation, and I have been able, with these means, to restore a paralyzed limb, and greatly to alleviate cases of Chronic Rheumatism. But I must pass to other features of disease where Galvanism has proved itself one of the most efficient and successful agents in our power. I refer to the class of Uterine Affections. Next to Paralysis, I know of no family of disease which baffles all the resources of medical art, and all the skill of consummate wisdom, more than the class before us. To be thrown, in a moment of time, under all the dread responsibility of preserving human life in one of its most interesting and fearful periods—that of parturition—to feel that a slight omission or a trifling commission, or nature herself, may place our patient in jeopardy which human power cannot control, brings a shudder over the stoutest heart.

In such an emergency, we hail with delight any means that promises to extricate us from even a possible dilemma, and no *one* item, I believe, has proved more of a specific than Electro-Galvinism for many conditions and disorders of the uterine system.

In inertia of the womb, either before labor, when the vital fluid is steadily passing away, or after labor, when life's flood-gates seem to have been opened, and the blood gushes in torrents, nothing has ever been found to check this condition, to close up these living streams, and produce tonic and periodic contraction of this organ like the agent of which I speak.

Take another condition—one enfeebled by lucorrhœal discharge, brought on by prolapsus, extending a miserable existence, exsanguinated, depressed, and irritable, I believe there is no panacea, no iron tonic or bitter specific, no pessary or supporter able to restore the lost vigor and enfeebled condition, like the wholesome stimulus of Galvanism.

So, too, in Amenorhœa, or suppression, you find young women whose friends talk of their going into a decline; the rosebud of health has been exchanged for the pallid, listless, tallowy look of Chlorosis, but a few shocks from the galvanic battery, at the appropriate time, along the nerves distributed to this organ, will do more to set the course of nature right, and mantle the cheek again with crimson, than the best assortment of drugs most assiduously applied for a twelvemonth.

Bo you ask again why this wonderful effect is produced in these cases? Because you strike at the seat of difficulty. You go to work at the cause; and having restored tone and function to the organ which occasioned the mischief, a state of health ensues. I know not why more of these cases have not been reported in the journals, but I was told at the dispensaries in New York city, that it was common for young women, emigrants, after a long sea voyage, to be troubled with suppression, and nothing had ever succeeded in removing the same, like Electro-Magnetism. I have used it and known of its application in several cases, and always with good results.

In part 13, of Braithwaite, I think, Mr. Dorsington relates three cases of Uterine Inertia, and sums up as follows :—

"That it is a very powerful remedy, there can be no doubt in the minds of those who have seen it tried, and that the uterus will respond to its application, whilst the general system is completely prostrated, is equally certain."

At the Rotunda Lying-in Hospital, Dublin, Mr. Clarke reports two cases of lingering labor from Uterine Inertia, in one of which the membranes had been ruptured 49 hours, and the other labor had lasted 60 hours, when the electro-magnetic current was employed with perfect success.

In all torpid conditions, where the vital powers seem disposed to flag, the stimulus of the galvanic machine is most favorable and salutary. I knew an old gentleman, upwards of 70 years of age, who used the instrument daily, for the warmth and stimulus which it afforded. If the galvanic current was able to digest the parsley in the stomachs of rabbits, as mentioned among our experiments after division of the par vagum, we would justly infer that in debilitated condition of that organ, in most forms of chronic Dyspepsia, at least some degree of this stimulus would be highly useful, and such it has proved to be, though but one case has ever been fairly tested in my own hands.

In regard to its relations to surgery, I cannot better present its claims to our consideration, than by using the language of Druitt, in his valuable work. Speaking of Electricity and Galvanism he says :—"Although these powerful agents have been by turns overrated and decried, and have lost much of their therapeutical reputation, through having been resorted to as the last desperate remedy, in cases where it was irrational to expect benefit from them, still no one who knows how to use them can doubt their efficacy.

"In certain cases of defective circulation and nervous influence—when the thigh is weak and benumbed with Sciatica; in cases of atrophy of the extremities after Fever, when the extensors are paralyzed from long disuse, as after disease of joints; in deficient menstruation; in Dyspnœa from weakness of the sotmach: in loss of voice, from relaxation of the mucous membrane of the fauces; in hysterical neuralgia, and in other cases of nervous pain, unattended with increased vascularity. they may be resorted to with every prospect of benefit;" and adds the most convenient apparatus seems to be a single battery, with a coil, and an apparatus for giving a stream of gentle shocks. Whether the indolent tumors which sometimes develope themselves in the scrofulous diathesis would be benefitted, I do not know, but reasoning from analogy, we are inclined to think Galvanism would be highly useful, and after protracted illness and convalescence, if anything would assist to arouse the dormant energies to their wonted life, this agent must be among the most certain and powerful. In cases of drowning, I believe this agent to be invaluable.

In its administration, "the feelings of the patient must be our guide, as to the strength of the charge in each particular case. Some will sustain with impunity, shocks which would be distressing and injurious to others."—[Ency, p. 297.

In the machine of which we are speaking, the small piston of iron rods graduates the amount with the greatest ease. In ordinary cases, 10 or 15 minutes, once or twice a-day, is as much as is necessary.

The application of the poles, to one familiar with the anatomy of the system, is not difficult, as you have only to place one over the root or trunk of the nerve, and the other over the affected part or the extremities of the same nerve.

In taking up a subject of this kind, entirely separate and disconnected from *all* others, many might infer that undue weight and unmerited importance was attached to it—that more force and power was given to this as a medicinal agent, than experience and facts would sustain. But I disavow any intention to distort the real worth of this as a curative means, or to detract from or neglect any other remedy whatever. I am not a "hobby" man. I believe, as I said in the outset, in the liberty and power of medical men selecting any and every agent from whatever source it may spring, so it stands the test of experience, and Electro-Magnetism, especially, I think one of those remedies which can oftentimes be brought in with other treatment, with the greatest advantage. I selected this topic in part to gratify my own curiosity, that I might better satisfy myself in regard to the powers and uses of this remedy, and also to direct the attention of the profession to this interesting subject, that still further advances might be achieved, new facts developed, suffering alleviated, and the world enlightened.

